# RUNX2 and LAMC2: promising pancreatic cancer biomarkers identified by an integrative data mining of pancreatic adenocarcinoma tissues

**DOI:** 10.18632/aging.203589

**Published:** 2021-10-04

**Authors:** Guihua Jin, Qingqing Ruan, Fugen Shangguan, Linhua Lan

**Affiliations:** 1 Key Laboratory of Diagnosis and Treatment of Severe Hepato-Pancreatic Diseases of Zhejiang Province, The First Affiliated Hospital of Wenzhou Medical University, Wenzhou 325000, China

**Keywords:** pancreatic carcinoma, bioinformatics analysis, biomarkers, LAMC2, RUNX2

## Abstract

Pancreatic carcinoma (PC) is a severe disease associated with high mortality. Although strategies for cancer therapy have made great progress, outcomes of pancreatic carcinoma patients remain extremely poor. Therefore, it is urgent to find novel biomarkers and therapeutic targets. To identify biomarkers for early diagnosis and therapy, three mRNA microarray datasets and two miRNA datasets were selected, and combinative analysis was performed by GEO2R. Functional and pathway enrichment analysis were performed using DAVID database. MiRTarBase, miRWalk and Diana Tools were used to get key genes. TCGA, HPA and western blotting were used to verify diagnostic and prognostic value of key genes. By integrating mRNA and miRNA expression profiles, we identified 114 differentially expressed genes and 114 differentially expressed miRNAs, respectively. Then, three overlapping key genes, RUNX2, LAMC2 and FBXO32, were found. Their protein levels in pancreatic tissue from PC patients and normal people were analyzed by immunohistochemical staining and western blotting. RUNX2 showed a potential property to identify PC. Aberrant over-expression of LAMC2 was associated with poor prognosis of PC patients, tumor status and subtypes. In summary, our current study identified that RUNX2 and LAMC2 may be promising targets for early diagnosis and therapy of PC patients.

## INTRODUCTION

Pancreatic carcinoma (PC) is one of the most malignant types of cancer in the world. Although considerable advancements in diagnostic and pharmacological therapy have been achieved, the 5-year overall survival (OS) rate of PC is still less than 9% [1]. Thus, novel diagnostic and prognostic biomarkers are urgently needed to improve individualized survival.

Microarray is a high throughput technique used to analyze genetic changes. It has revolutionized studies on RNA and DNA and has also been extensively implemented as an efficient tool for the exploration of genes and biological processes in human diseases. Several studies have reported the differentially expressed genes (DEGs) and differentially expressed microRNA (DEMs) of PC in recent years [2–4]. However, there remain unanswered questions about the interaction between DEGs and DEMs during the progression of PC. And it’s necessary for uncovering the real target genes.

In this work, we applied three mRNA profiling datasets (GSE62165 [5], GSE15471 [6] and GSE32676 [7]) and two microRNA (miRNA) profiling datasets (GSE24279 [8] and GSE32678 [7]), which were downloaded from the Gene Expression Omnibus (GEO) database, to obtain DEGs and DEMs between PC and normal tissue samples. Subsequently, DEMs targeted genes (DEMTGs) were predicted, and by overlapping analysis between DEMTGs and DEGs to filter potential genes and microRNAs (miRNAs). We further clarified differential expression of key genes through The Cancer Genome Atlas (TCGA) database. Finally, the diagnostic and prognostic values of these key genes and miRNAs were evaluated and predicted by receiver operating characteristic (ROC) and survival analysis.

## MATERIALS AND METHODS

### Data collection and DEGs/DEMs/tDEGs/tDEMs analysis

Gene expression profiles were downloaded from the GEO public database (http://www.ncbi.nlm.nih.gov/geo/) and the inclusion criteria were as follows: (1) pancreatic cancer, (2) January 1, 2009 to December 31, 2019, (3) homo sapiens, (4) tissue, (5) sample count exceeds 30, (6) mRNA or miRNA expression data available. As a result, a total of 5 datasets were selected, which included GSE62165, GSE15471, GSE24279, GSE32676 and GSE32678.

The GEO2R web tool was used to screen for DEGs and DEMs between the PC and normal tissue samples in each dataset. The log2FoldChange (logFC) was calculated and the P-values were adjusted to correct for the occurrence of false-positive results by using the default method. Then, P-value < 0.05 and |log2FC| > 1.5 as the cut-off criteria for significant DEGs, and P-value < 0.05 and |log2FC| > 0.5 for significant DEMs was settled. Subsequently, volcano plots of DEGs and DEMs were used to quickly identify differences and Venn analysis was performed to get overlapping up or down regulated DEGs/DEMs in all mRNA/miRNA datasets, respectively [9]. By adding common shared up regulated DEGs/DEMs with down regulated DEGs/DEMs, obtained datasets were named as tDEGs and tDEMs in this study.

### Enrichment analysis and key genes acquisition

Target genes of tDEMs (tDEMTGs) were predicted by the datasets which downloaded from miRTarBase (http://miRTarBase.cuhk.edu.cn/), miRWalk (http://mirwalk.umm.uni-heidelberg.de/) and Diana Tools (http://diana.imis.athena-innovation.gr/DianaTools/index.php) [10–12]. Gene ontology (GO) and Kyoto Encyclopedia of Genes and Genomes (KEGG) were applied for the potential relevant functional annotation and pathway enrichment analysis of tDEMTGs and tDEGs were analyzed by the Database for Annotation Visualization and Integrated Discovery (DAVID, https://david.ncifcrf.gov/) [13]. Then, tDEMTGs were aligned with tDEGs to obtain the intersection termed “key genes” for further analysis. Additionally, tDEMs which were relevant to key gene regulation termed as “candidate miRNAs”.

### Survival analysis

To evaluate the prognostic value of key genes and miRNAs, the overall survival (OS) of PC patients was investigated based on the online database, Kaplan-Meier Plotter (http://kmplot.com/analysis/index.php) [14]. High- and low-level cohorts of the indicated genes were set according to the median value of gene expressions. The OS plot was obtained with the hazard ratio (HR), the 95% confidence interval (CI) and logrank P-value information.

### Key gene expression analysis in TCGA and genotype-tissue expression (GTEx) tissues

Gene Expression Profiling Interactive Analysis (GEPIA [15], http://gepia.cancer-pku.cn/) is an interactive web server for estimating the RNA-Seq expression data from the TCGA (https://www.cancer.gov/about-nci/organization/ccg/research/structural-genomics/tcga) and GTEx (https://www.gtexportal.org/) databases. The expression of key genes was analyzed in various tumor and non-tumor tissues using GEPIA, and the comparison of the key gene expression at different stages of PC was also performed. The clinical information of patients was also downloaded from TCGA for further data validation.

### Data validation

Blood and saliva GEO data verification: We investigated a validation by comparing the mRNA standardized values of key genes in five independent GEO datasets. Four of them were blood samples (GSE74629 [16], GSE49641 [17], GSE49515 [18] and GSE15932) and the last one was saliva samples (GSE14245 [19]).

Immunohistochemistry (IHC) verification: The IHC staining images of PC and normal tissue, which obtained from the Human Protein Atlas [20] (HPA, https://www.proteinatlas.org), were used as validation of key genes. The staining, intensity, quantity and location of IHC images present in HPA were calculated for each gene. (For the more details about statistical methods of the IHC images, the reader is referred to the web page https://www.proteinatlas.org/about/assays+annotation#ih_annotation).

Western blotting (WB) verification: Sixteen pancreatic tissue samples from PC patients were obtained by surgical resection and further divided into tumor group and paracancerous group (normal). The study was approved by the Institutional Ethics Committee of Wenzhou Medical University and written informed consent was obtained from each patient before their enrollment. WB protocol was performed according to our previously described procedures [21]. The following primary antibodies were used: RUNX2 (ABclonal, cat. no. A2851), LAMC2 (ABclonal, cat. no. A1869), AKT (Cell signaling Technology, cat. no. #4685), p-AKT (Cell signaling Technology, cat. no. #4060), p-c-Raf (Cell signaling Technology, cat. no. #9421), p-MEK1/2 (Cell signaling Technology, cat. no. #9154), p-ERK1/2 (Cell signaling Technology, cat. no. #4370), p-p90RSK (Cell signaling Technology, cat. no. #9346), p-MSK1 (Cell signaling Technology, cat. no. #9595), and β-Actin (Abmart, cat. no. P30002). The protein expression levels were quantified by Image J software.

### Statistical analysis

The discriminative ability of key genes and miRNAs in the GEO datasets was calculated by ROC analysis, the pROC [22] R package was performed in R 3.6.2 (http://www.R-project.org/). The area under the curve (AUC), 95% CI and P-value for assessing the predictive accuracy and specificity of ROC were calculated by SPSS version 23.0 (IBM, Chicago, IL, USA). All scatter and bar plots were generated by Graph Pad Prism 7 Software (GraphPad Software, Inc.). Comparisons among means were performed by ANOVA. P-value < 0.05 was assessed as statistically significant.

## RESULTS

### Initial identification of tDEGs, tDEMs and tDEMTGs in pancreatic cancer

**Figure 1:**
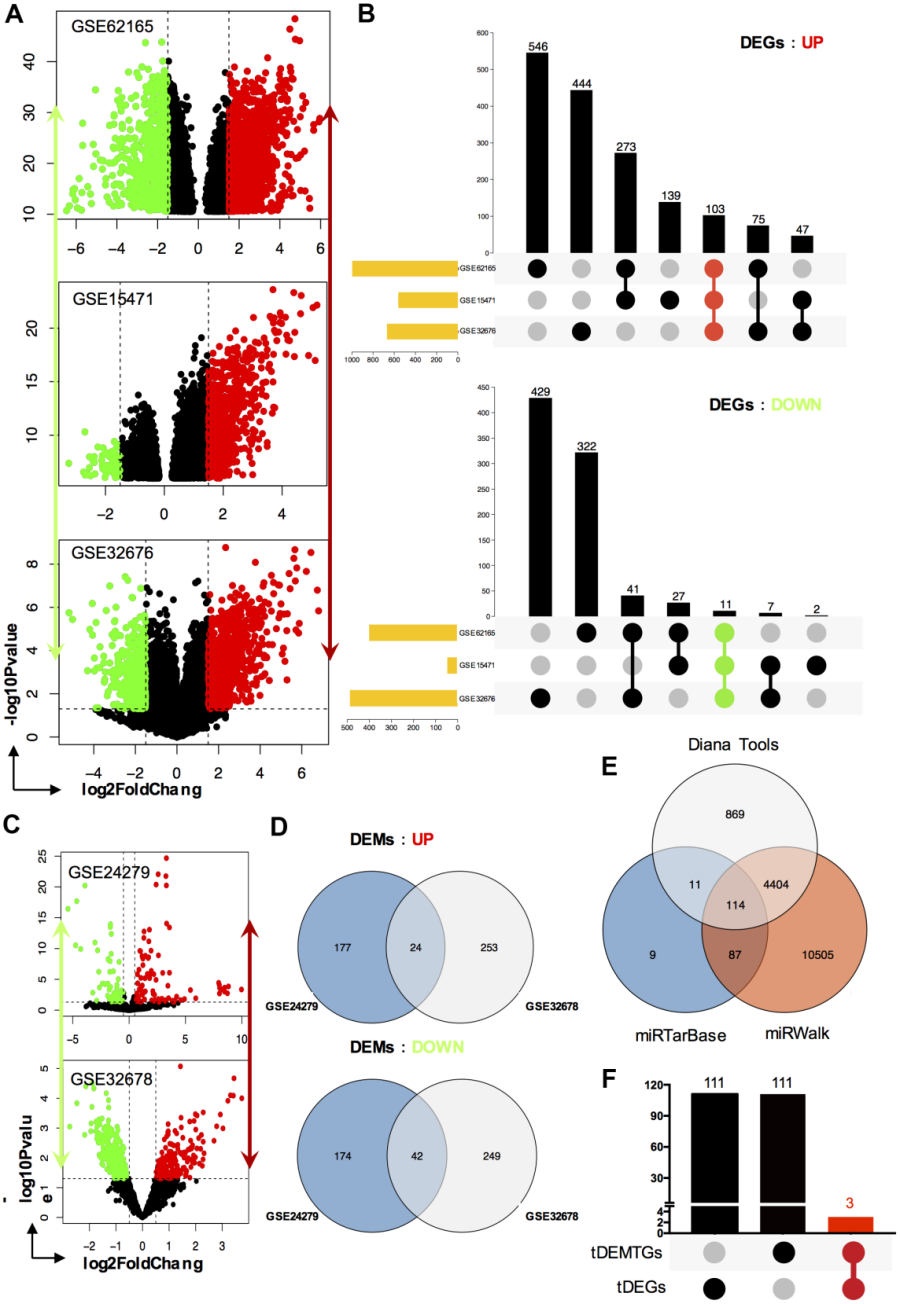
**Identification of DEGs, DEMs and key genes.** (**A**) The volcano plot of mRNA GEO datasets GSE62165, GSE15471 and GSE32676 respectively; (**B**) Venn diagram of up-regulated DEGs and down-regulated DEGs set from GSE62165, GSE15471 and GSE32676 datasets; (**C**) The volcano plot of miRNA GEO datasets GSE24279 and GSE32678 respectively; (**D**) Venn diagram of up-regulated DEMs and down-regulated DEMs set from GSE24279 and GSE32678 datasets; (**E**) The target genes, tDEMTGs, of 66 consistent DEMs (tDEMs) were predicted by miRTarBase, miRWalk and Diana Tools databases. (**F**) Venn diagram of tDEMTGs and tDEGs to get key genes.

To find key genes that were differentially expressed between PC patients and healthy people, we decided to filter them both ways: mRNA and miRNA targeted method. Firstly, we selected three expression array profiling datasets (GSE62165, GSE15471 and GSE32676) and two non-coding RNA array profiling datasets (GSE24279 and GSE32678) from GEO database. As for mRNA, 1398, 607 and 1157 DEGs were extracted from GSE62165, GSE15471 and GSE32676. Among them, there were 997, 562, 669 (103 shared genes) up-regulated and 401, 45, 488 (11 shared genes) down-regulated DEGs were identified. Taking advantage of Venn analysis in up or down regulated DEGs respectively, we captured 114 total-shared DEGs (tDEGs). Among them, there were 103 up-regulated and 11 down-regulated genes (Figure 1A, 1B). As for miRNA, the same operations were also performed. 417 and 569 DEMs were extracted from GSE24279 and GSE32678. Two miRNA datasets shared 66 total DEMs (tDEMs), including 24 up-regulated and 42 down-regulated miRNAs (Figure 1C, 1D).

Target genes for tDEMs were predicted by miRTarBase, miRWalk and Diana Tools databases. Then 114 consistent genes, which we termed as tDEMTGs, were found by Venn’s analysis (Figure 1E).

### GO and pathway enrichment analysis

**Figure 2:**
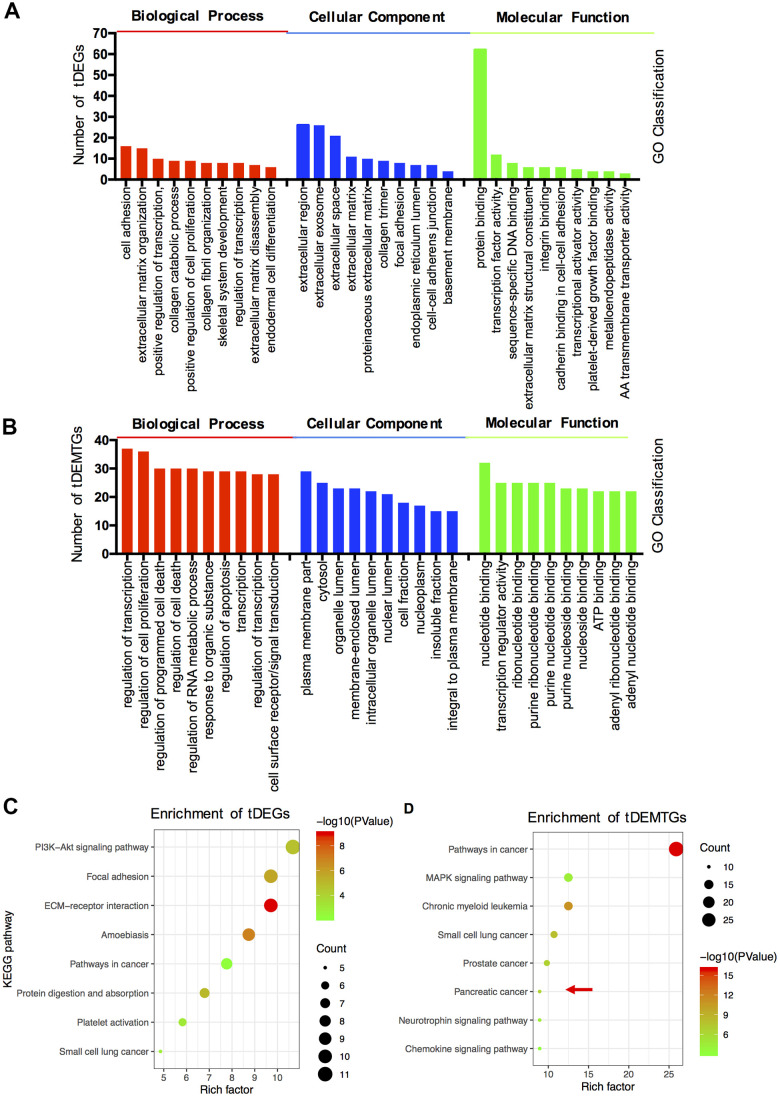
**The functional and pathway enrichment analysis of tDEGs and tDEMTGs.** (**A**) Biological process, cellular component and molecular function analysis for tDEGs, top 10 of each GO classification were listed; (**B**) Biological process, cellular component and molecular function analysis for tDEMTGs; (**C**) KEGG pathway enrichment analysis for tDEGs, top 8 were listed; (**D**) KEGG pathway enrichment analysis for tDEMTGs.

The GO term and KEGG pathway analysis were performed via DAVID. First, the results of the GO enrichment analysis varied a lot between tDEGs and tDEMTGs (Figure 2A, 2B). Biological process (BP) analysis of GO showed tDEGs were significantly enriched in extracellular matrix associated part, such as cell adhesion, extracellular matrix organization and disassembly, collagen catabolism and organization. As for tDEMTGs, top 2 clusters of genes were enriched in the regulation of transcription and proliferation, which showed a similar result in tDEGs, but other genes were mainly responsible for the regulation of cell death associated part. For GO cell component (CC) analysis, the tDEGs were significantly enriched in extracellular part, such as extracellular region, extracellular exosome, extracellular space, extracellular matrix and proteinaceous extracellular matrix. However, the results of tDEMTGs were focused on plasma membrane part, cytosol, organelle lumen, membrane-enclosed lumen and intracellular organelle lumen. The same differences also showed in molecular function (MF) analysis, tDEGs were mainly enriched in protein binding, but tDEMTGs were enriched in nucleotide binding, transcription regulator activity, ribonucleotide binding, purine ribonucleotide binding and purine nucleotide binding.

Additionally, the differences between tDEGs and tDEMTGs were also confirmed by KEGG pathway analysis. The result disclosed that tDEGs were involved in PI3K/Akt signaling pathway, focal adhesion, ECM-receptor interaction, amoebiasis and pathways in cancer (Figure 2C). However, tDEMTGs were significantly enriched in pathways in cancer and partial enriched in pancreatic cancer (Figure 2D).

### Identification of key genes and miRNAs

To identify potential key genes, tDEMTGs were aligned with tDEGs to get the cross-section, which we termed as key genes for further analysis (Figure 1F). Importantly, the result showed that runt related transcription factor 2 (RUNX2), laminin subunit gamma-2 (LAMC2) and F-box protein 32 (FBXO32) were commonly shared and then listed in Table 1. Subsequently, we analyzed candidate miRNAs involved in the regulation of key genes from 66 tDEMs, and found 16 up-regulated and 19 down-regulated key miRNAs (Table 2).

**Table 1:** The differential expression of key genes in different GEO datasets.

**Gene**	**Datasets**	**ID**	**LogFC**	**P.Val**	**Expression**
RUNX2	GSE62165	11725180_a_at	3.7	3.10E-29	UP
	GSE15471	232231_at	2.93	9.52E-11	UP
	GSE32688(mRNA)	232231_at	1.69	0.1046045	UP
LAMC2	GSE62165	11737325_a_at	3.49	4.80E-25	UP
		11743514_a_at	4.09	7.56E-26	UP
		11743516_s_at	4.29	1.49E-26	UP
		11743515_s_at	4.35	1.06E-28	UP
		11747923_s_at	4.37	7.93E-31	UP
	GSE15471	207517_at	1.60	3.83E-09	UP
		202267_at	2.76	1.76E-13	UP
	GSE32688(mRNA)	202267_at	4.93	0.0002728	UP
		207517_at	2.25	0.0038994	UP
FBXO32	GSE62165 GSE15471	11719394_a_at	2.88	3.83E-22	UP
		225803_at	1.72	9.65E-14	UP
		241762_at	1.83	2.64E-11	UP
		225328_at	1.91	5.28E-12	UP
	GSE32688(mRNA)	241763_s_at	2.44	0.0095169	UP
		241762_at	2.32	0.0215962	UP
		225803_at	1.88	0.0176426	UP
		225345_s_at	1.56	0.0702839	UP

**Table 2:** The differential expression of candidate miRNAs in different GEO datasets.

**Target gene**	**miRNA**	**Datasets**	**LogFC**	**Expression**
RUNX2	hsa-miR-519c-3p	GSE32678	0.531577	UP
		GSE24279	3.2867924	
	hsa-miR-876-3p	GSE32678	0.755198	UP
		GSE24279	9.9730133	
	hsa-miR-1265	GSE32678	0.883218	UP
		GSE24279	0.6795887	
	hsa-miR-92a-2-5p	GSE32678	-1.231335	DOWN
		GSE24279	-0.7331099	
	hsa-miR-193b-5p	GSE32678	0.6020578	DOWN
		GSE24279	-1.218209	
	hsa-miR-517-5p	GSE32678	-0.606971	DOWN
		GSE24279	-1.2242733	
	hsa-miR-488-3p	GSE32678	-1.145003	DOWN
		GSE24279	-1.145003	
RUNX2/FBXO32	hsa-miR-23b-3p	GSE32678	1.214636	UP
		GSE24279	0.7358448	
	hsa-miR-221-5p	GSE32678	1.277623	UP
		GSE24279	1.8034373	
	hsa-miR-199a-5p	GSE32678	1.240204	UP
		GSE24279	1.6889355	
	hsa-miR-376a-5p	GSE32678	0.546421	UP
		GSE24279	2.3186641	
	hsa-miR-1825	GSE32678	1.225039	UP
		GSE24279	4.0684489	
	hsa-miR-1227-3p	GSE32678	0.687478	UP
		GSE24279	3.0619077	
	hsa-miR-198	GSE32678	-1.004687	DOWN
		GSE24279	-0.6024842	
	hsa-miR-937-3p	GSE32678	-0.768939	DOWN
		GSE24279	-1.2399531	
FBXO32	hsa-miR-345-5p	GSE32678	0.821379	UP
		GSE24279	7.9474776	
	hsa-miR-1233-3p	GSE32678	0.528843	UP
		GSE24279	1.0034567	
	hsa-miR-1289	GSE32678	0.583983	UP
		GSE24279	0.5373088	
	hsa-miR-621	GSE32678	0.962951	UP
		GSE24279	0.9769538	
	hsa-miR-1248	GSE32678	1.377747	UP
		GSE24279	1.0123159	
	hsa-miR-30b-3p	GSE32678	-0.705179	DOWN
		GSE24279	-0.7012921	
	hsa-miR-373-5p	GSE32678	-0.943119	DOWN
		GSE24279	-1.5962925	
	hsa-miR-492	GSE32678	-0.665303	DOWN
		GSE24279	-1.1011978	
	hsa-miR-510-5p	GSE32678	-1.069006	DOWN
		GSE24279	-2.499187	
	hsa-miR-575	GSE32678	-0.77259	DOWN
		GSE24279	-0.5620953	
	hsa-miR-604	GSE32678	-0.508662	DOWN
		GSE24279	-1.2474484	
	hsa-miR-611	GSE32678	-1.159661	DOWN
		GSE24279	-1.6342346	
	hsa-miR-617	GSE32678	-0.816815	DOWN
		GSE24279	-1.5017728	
	hsa-miR-921	GSE32678	-0.90506	DOWN
		GSE24279	-1.9104782	
	hsa-miR-520d-5p	GSE32678	-0.660262	DOWN
		GSE24279	-0.6523876	
FBOX32/LAMC2	hsa-miR-199b-5p	GSE32678	1.544751	UP
		GSE24279	1.0106128	
LAMC2	hsa-miR-588	GSE32678	0.526075	UP
		GSE24279	4.3557666	
	hsa-miR-891a-5p	GSE32678	-0.713461	DOWN
		GSE24279	-1.3790634	
	hsa-miR-622	GSE32678	-0.961764	DOWN
		GSE24279	-1.1362947	
LAMC2/RUNX2	hsa-miR-628-3p	GSE32678	-0.64033	DOWN
		GSE24279	-0.9205919	

### Diagnostic and prognostic values of candidate miRNAs

**Figure 3:**
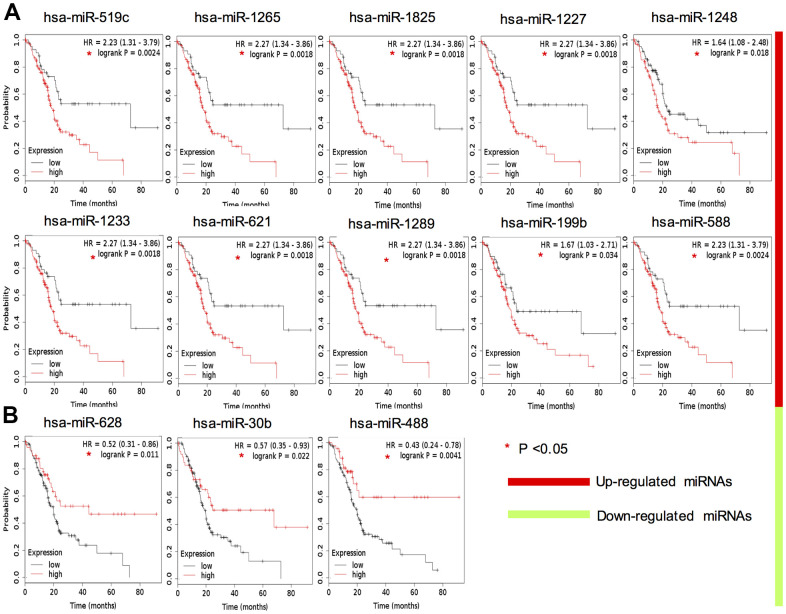
**Survival analysis of 13 key DEMs expression in TCGA PAAD cohort.** Red color indicates upregulation (**A**), whereas green color indicates that the miRNA is downregulated (**B**).

To test the clinical applicability of the candidate miRNAs, we investigated their diagnostic significance through ROC curve analysis (Supplementary Figure 1A). However, the mean AUC values of them were all lower than 0.85 (Supplementary Figure 1B). To further evaluate their prognostic value, survival analysis was applied and the results showed 10 up-regulated key miRNAs indicated a significantly lower OS rate: hsa-miR-519c-3p, hsa-miR-1265, hsa-miR-1825, hsa-miR-1227-3p, hsa-miR-1233-3p, hsa-miR-1289, hsa-miR-621, hsa-miR-1248, hsa-miR-199b-5p and hsa-miR-588 (Figure 3A). Correspondingly, 3 down-regulated candidate miRNAs were positively related with OS rate: hsa-miR-488-3p, hsa-miR-30b-3p and hsa-miR-628-3p (Figure 3B). These data indicated that these 13 candidate miRNAs could be potential prognostic markers for clinical application.

### Diagnostic and prognostic values of key genes

**Figure 4:**
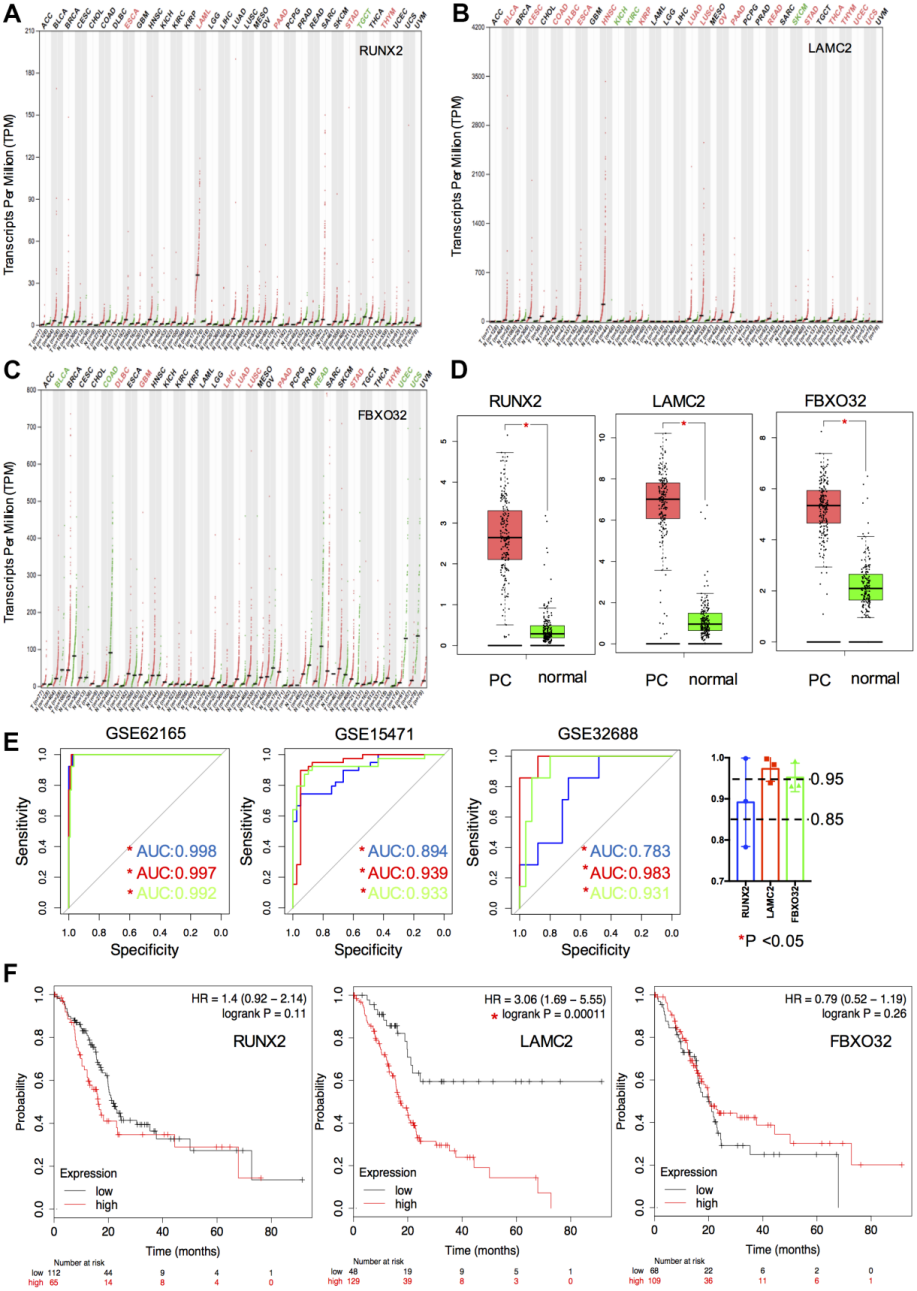
**Expression, ROC and survival analysis of key genes.** (**A**–**C**) Differential expression of key genes (RUNX2, LAMC2 and FXBO32) in various tumor and non-tumor tissues. (**D**) Differential expression of key genes in PC patients and normal controls. (**E**) ROC curve of key genes in GSE62165, GSE15471 and GSE32688. (**F**) Survival analysis of key genes in TCGA PAAD cohort.

We then compared the transcriptional levels of key genes in several cancers with those of normal samples using TCGA database via GEPIA (Figure 4A–4C). The mRNA expression levels of RUNX2, LAMC2 and FBXO32 showed significant differences between cancer and adjacent non-cancerous tissues especially in pancreatic adenocarcinoma (PAAD, Figure 4D). ROC and survival analysis were also performed to ensure the application prospect of key genes. All the three key genes indicated a good discriminating ability, of which the mean AUC values of each key gene in three GEO datasets were all greater than 0.85 (Figure 4E). Furthermore, the mean AUC values of LAMC2 and FBXO32 were both greater than 0.95 which indicated that these two molecules might be promising biomarkers for PC diagnosis. The results of survival analysis showed that only the LAMC2 expression level affected OS rate significantly (HR=3.06, P=0.00011, Figure 4F). It suggested that LAMC2 could be an important molecule which participate in the development of PC and also act as a potential prognostic biomarker.

### Verification of key genes

In addition to high accuracy and specificity, the diagnostic methods should also be painless and woundless. Blood and saliva are easily obtained specimens that we can get with little body damage. Thus, we verified the diagnostic possibility of key genes in 4 blood GEO datasets (GSE74629, GSE49641, GSE49515 and GSE15932) and 1 saliva GEO dataset (GSE14245). Interestingly, we found only RUNX2 had significant reduction compared with normal controls in saliva and blood cells (GSE14245 and GSE49641), which suggested that RUNX2 could be considered as one of promising candidate biomarkers of PC. LAMC2 and FBXO32 had no significant differences between the control and PC samples.

**Figure 5:**
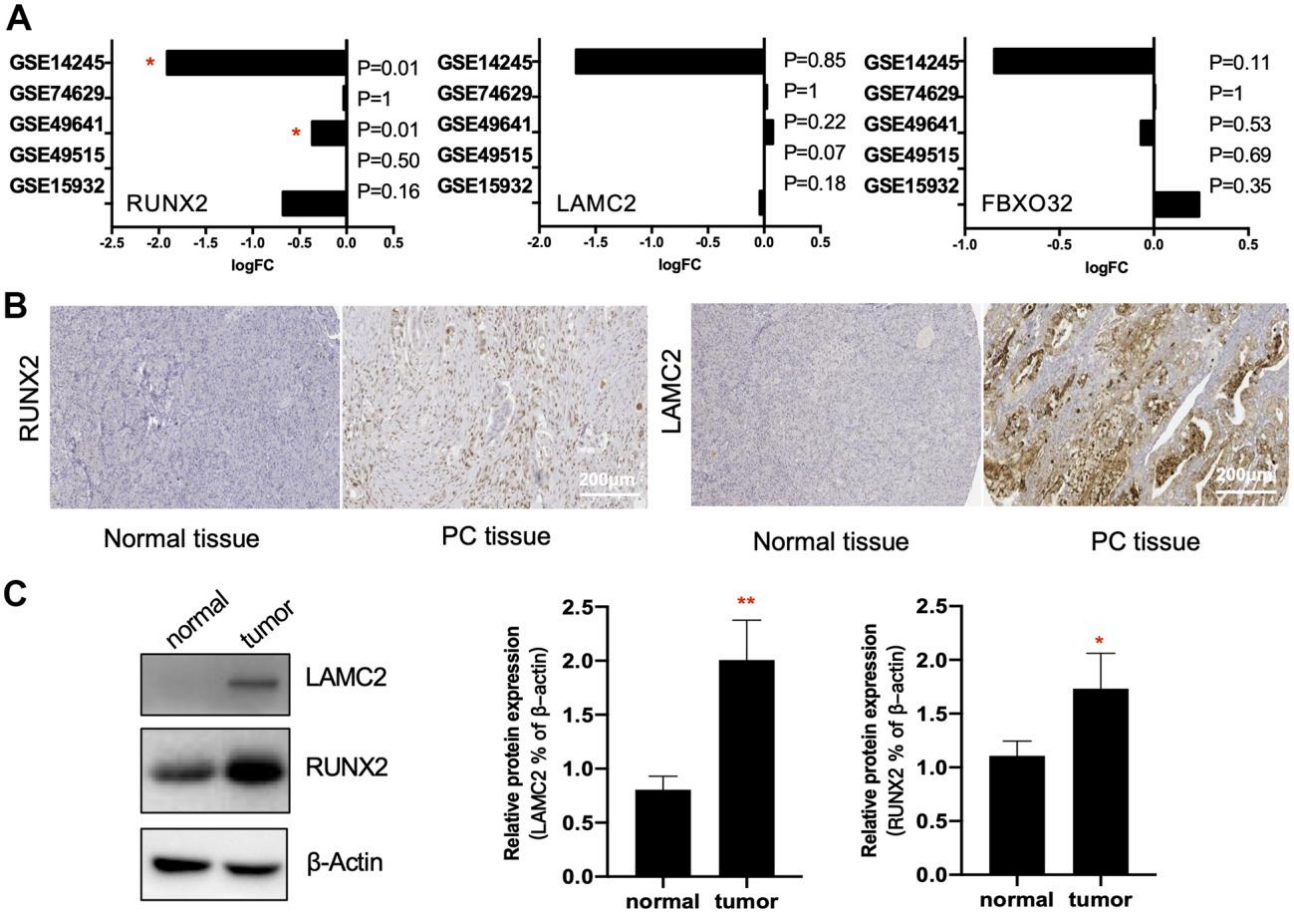
**Verification of key genes.** (**A**) RUNX2, LAMC2 and FXBO32 expression levels (Log2FoldChange value) in GSE14245, GSE74629, GSE49641, GSE49515 and GSE15932. (**B**) Representative immunohistochemistry staining of RUNX2 and LAMC2 in pancreatic ductal adenocarcinoma (PC tissue) and control pancreatic tissue (Normal tissue) in Human Protein Atlas (HPA). Scales bars represent 200 μm. (**C**) Western blot analysis for RUNX2 and LAMC2 expression proteins in 16 paired samples from PC patients.

Then, to further clarify the expression of RUNX2 and LAMC2 in PC tissues, the results of IHC images of these two genes in HPA were explored. (Figure 5B and Supplementary Figure 2). First, the expression of LAMC2 was consistent with previous analysis. The staining of anti-LAMC2 antibodies were all above medium level in PC patients, which were barely detected in normal pancreas tissues. Although staining quantity were varied (5.5/12 were >75%, 2.5/12 were 75%-25% and 4/12 were <25% stained), almost all of their staining intensity were strong. However, the result of RUNX2 was not corroborated with predicted. Though a slight up-regulation of RUNX2 could be found in some samples (17%), most could not be detected. In addition, our immune blot results were consistent with IHC experiment (Figure 5C). These data indicated that LAMC2 may play a crucial role in the PC therapy.

**Figure 6:**
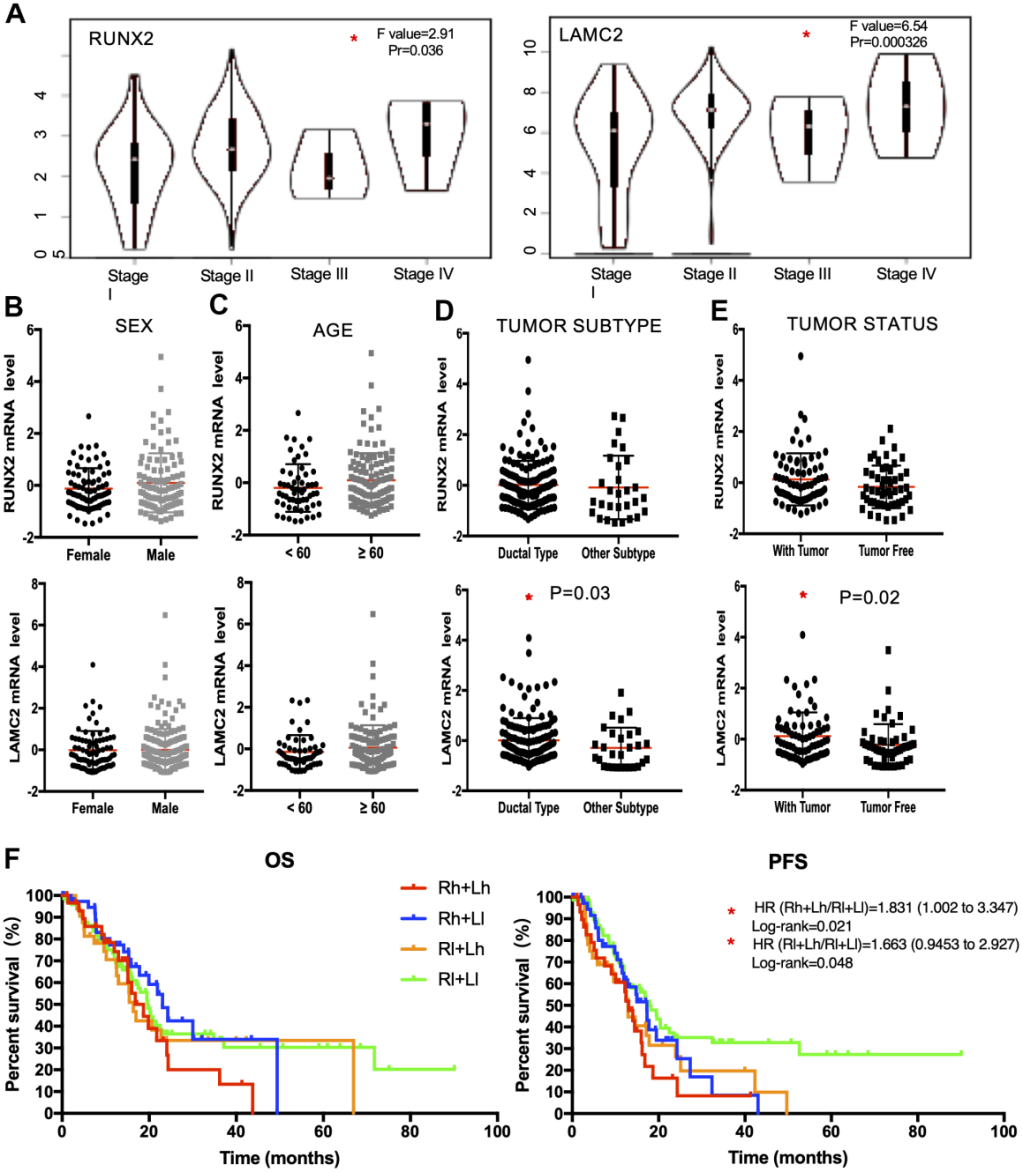
**The clinical application value of RUNX2 and LAMC2 expression.** (**A**) Expression levels of RUNX2 and LAMC2 in pancreatic cancer (PC) patients of different clinical stages. (**B**) Expression levels of RUNX2 and LAMC2 in different sex groups of PC patients. (**C**) Expression levels of RUNX2 and LAMC2 in different age groups of PC patients. (**D**) Expression levels of RUNX2 and LAMC2 in different tumor subtypes of PC patients. (**E**) Expression levels of RUNX2 and LAMC2in different tumor status groups of PC patients. (**F**) Survival analysis of combined RUNX2 and LAMC2 in TCGA PAAD cohort.

In a second validation study, we explored the expression of RUNX2 and LAMC2 in different clinical stage, sex, age, tumor subtype and personal tumor status groups (Figure 6A–6E). We found RUNX2 was only significantly up-regulated in advanced stage groups. However, LAMC2 were significantly elevated not only in advanced PC patients, but also in the ductal type PC group (P=0.03) and PC patients which with tumor group (P=0.02).

According to all the above evidence, we found LAMC2 could be the major factor influencing the median survival time of PC patients through combinatorial analysis of RUNX2 and LAMC2 (Figure 6F and Supplementary Tables 1, 2).

### LAMC2 and RUNX2 regulate PC cell growth and migration

**Figure 7:**
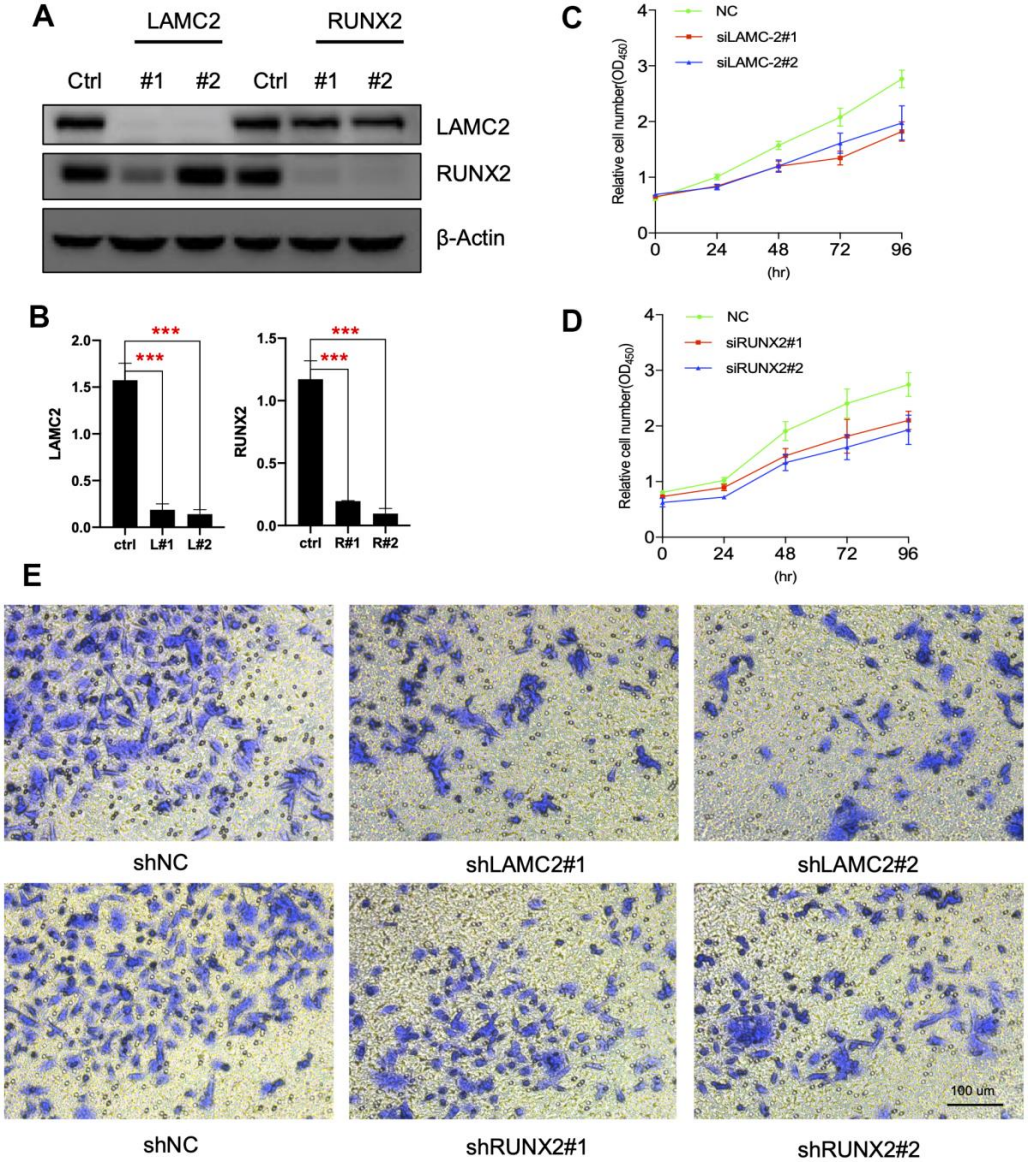
**LAMC2 and RUNX2 participate in PC cell growth and migration.** (**A**, **B**) ASPC-1 cells were transfected with control shRNA (NC) or two shRNAs of different sequences targeting LAMC2 or RUNX2 for 48 h; cell samples were collected and subjected to western blot analysis. (**C**, **D**) Cell growth curves of control, LAMC2- and RUNX2-depleted cells were measured by RTCA (Real Time Cellular Analysis). (**E**) Cell migration of control, LAMC2- and RUNX2-depleted cells were plotted by transwell assay.

**Figure 8:**
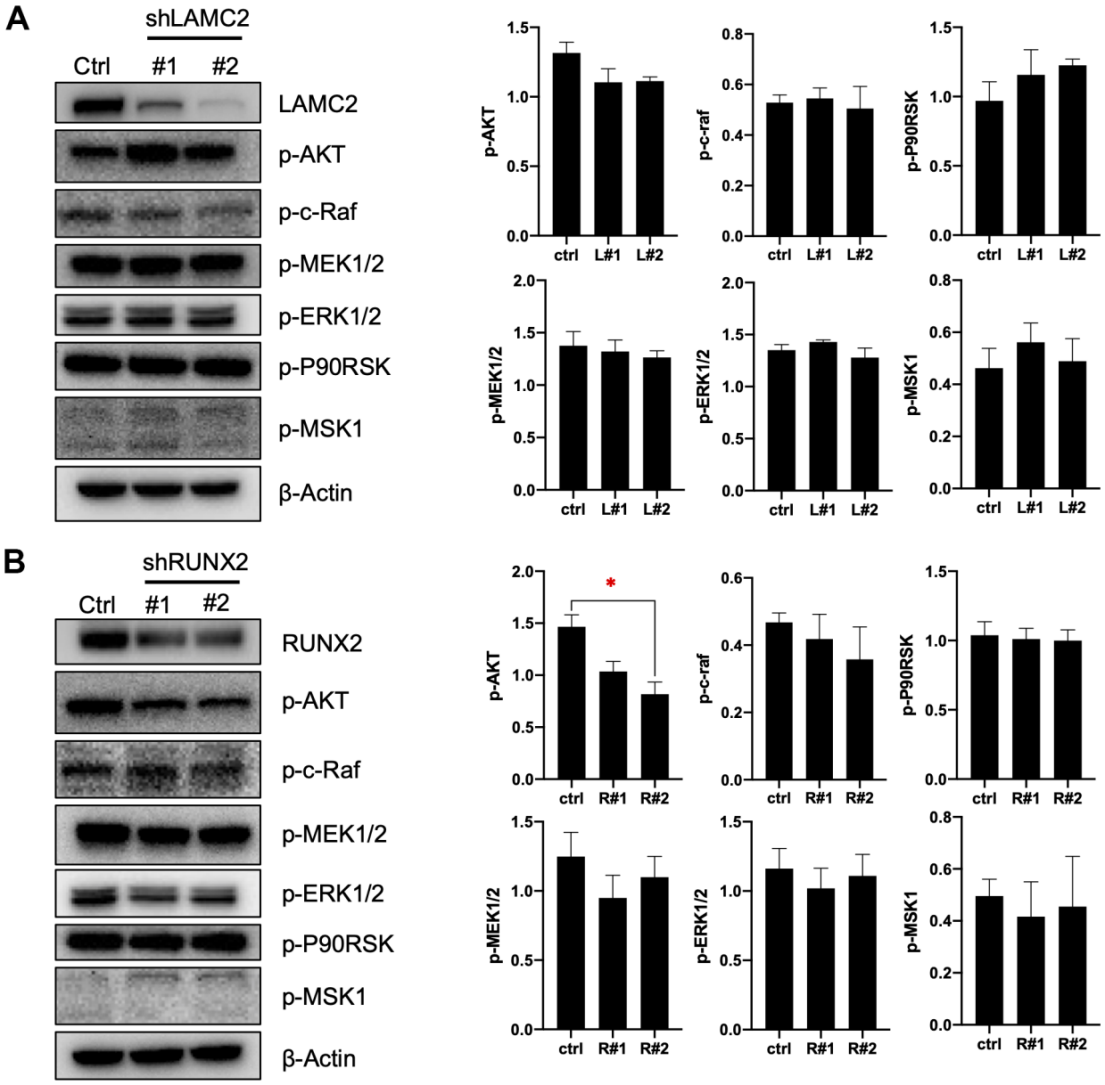
**LAMC2 and RUNX2 regulate PC cell growth and migration through PI3K/AKT and MAPK pathway.** (**A**, **B**) ASPC-1 cells were stably transfected with control shRNA (NC) or two shRNAs of different sequences targeting LAMC2 or RUNX2; cell samples were collected and subjected to Western blot analysis of p-AKT, p-c-Raf, p-MEK1/2, p-ERK1/2, p-P90RSK and p-MSK1. Actin was used as a loading control. The data expressed in right graphs represent the mean ± SEM (*p < 0.05).

For this, we knocked down LAMC2 or RUNX2 using shRNAs in ASPC-1 cells (Figure 7A, 7B) and assessed the subsequent cell growth (Figure 7C, 7D) and migration (Figure 7E). We found that knockdown of LAMC2 or RUNX2 both inhibited PC cell growth and migration significantly. Then, we measured changes of PI3K/AKT and MAPK pathways (Figure 8A, 8B) since their vital roles in PC progression (Figure 2C, 2D). Knockdown of RUNX2 significantly inhibited phosphorylation of AKT, which implied an important role of RUNX2/AKT in the progression of PC. Knockout LAMC2 had no significant effect on the PI3K/AKT and MAPK pathways, but significantly reduced the expression of vimentin which is up-regulated during epithelial-mesenchymal transition (Supplementary Figure 3).

## DISCUSSION

Recently, the increasing morbidity and mortality of PC has made it a serious challenge for human health. However, benefiting from the development of bioinformatics technology, it is much easier for us to identify the general genetic alterations in diseases now, which may shed light on the determination of hub targets for clinical utility. Studies of miRNA biology have expanded considerably since first discovery, and have been considered as attractive targets because of their crucial roles in modulation of gene expression under healthy, inflamed and carcinogenic pathological state. Therefore, analysing gene expression changes in combination with miRNAs is vital for identifying general diagnostic and prognostic biomarkers in cancer.

In the present study, we initially found 114 tDEGs and 114 tDEMs from GEO datasets respectively. By GO function and KEGG analysis, we gained a deep understanding of these genes attached to the initiation and progression of PC. Further, to study the general profiles of molecular alterations in PC, we then identified 3 key genes and 35 important miRNAs by aligning tDEMTGs with tDEGs. We investigated diagnostic and prognostic value of them by ROC and survival tests. And we also confirmed protein expression through IHC and WB assays. Based on the data from this study, we suggested that LAMC2 and RUNX2 could serve as potential biomarkers for the clinical use of PC in the future.

**Figure 9:**
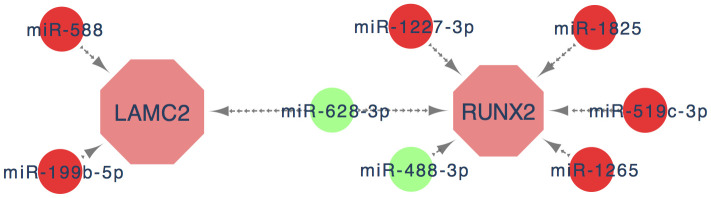
**The key genes and key miRNA interaction network.** Red nodes denote up-regulated key genes or miRNAs, while green nodes denote down-regulated key genes or miRNAs. The lines represent an interaction relationship between the nodes.

MiRNAs are a class of non-coding RNAs which can bond to 3'-untranslated region (3'-UTR) of targeted mRNA to regulate their protein expression levels [23]. Multiple studies have demonstrated that miRNAs participate in the management of all cancer hallmarks. It is generally believed that miRNAs could be important molecular tools for non-invasive diagnosis and prognosis of cancer. Therefore, it is of great importance to identify most commonly suitable DEGs which could be used as treatment targets or diagnostic markers by discussing the interaction of miRNAs and mRNAs. In this study, we finally obtained 8 key miRNAs from candidate miRNAs by further screening through ROC and survival analysis, which enriched miRNA profiles of PC and may help to highlight the diagnostic and therapeutic potential of the miRNAs cluster in PC (Figure 9).

RUNX2 belongs to RUNX family and is known as one of the main determinants of osteoblast differentiation and bone formation. Recent studies found RUNX2 was overexpressed in several tumor tissues and may play a vital role in tumor initiation, progression, invasion and metastasis [24–28]. This study demonstrated that although overexpression of RUNX2 was related to malignant behaviours, it didn't significantly affect the survival time of the PC patients which might be a bit inconsistent with previous research. Intriguingly, we found that RUNX2 presented a high diagnostic capability in PC. Histology is an invasive test that is inappropriate for the early diagnosis of PDAC, in terms of safety. Therefore, blood or body fluid tests can provide a good alternative method to assist clinicians with diagnosis. In contrast to the high expression of RUNX2 in pancreatic tissue, it was down-regulated in blood datasets, and its expression in peripheral blood mononuclear cell (PBMC, GSE49641) was significantly lower in PC group than in healthy control samples. Saliva samples (GSE14245) showed a similar result which suggests its applicability as a non-invasive diagnostic biomarker. However, there was no significant change in peripheral blood (GSE74629 and GSE15932). In addition, RUNX2 has been reported that could regulate many carcinogenesis genes and molecules in PC, including VEGF (vascular endothelial growth factor), matrix metalloproteinases and survivin [29]. Combined with the results of transcription factor prediction, we believed that the role of RUNX2 in PC progression might concentrate more on regulating the target genes, such as LAMC2.

LAMC2, a member of laminins family, is the main structural component of basement membranes and participates in a wide variety of biological processes including cell adhesion, differentiation, migration and metastasis [30, 31]. In this study, the result of the enrichment analysis indicated that LAMC2 was closely related with the process of cell adhesion, extracellular matrix organization/disassembly and positive regulation of cell proliferation. We then found that the expression of LAMC2 was elevated in PC tumor tissues through the GEO database, IHC and WB analysis. Additionally, for PC patients with high LAMC2 expression, the prognosis was poor. According to these data, we hypothesized that the expression of LAMC2 may lead to an increased risk of tumor recurrence. Pancreatic ductal adenocarcinoma (PDAC) accounts for most human PC cases (more than 95%) [32], we found that the expression of LAMC2 is mainly up-regulated in PDAC, which indicates that it is not only suitable for most PC patients, but may also help to identify the subtypes of PC. Because carbohydrate antigen 19-9 (CA19-9) which acts as the most commonly used PC marker is not accurate for the diagnosis of PDAC [33], combining with the good discriminating ability that LAMC2 showed in the AUC curve, we suggest that it may be a potential indicator in the auxiliary diagnosis of PC.

The PI3K/AKT and MAPK pathways are both important intracellular signal transduction cascades which could regulate cell growth and proliferation, survival and apoptosis, and mobility and invasion [34–36]. In tumorigenesis, dysregulation of PI3K/AKT and/or MAPK pathways occurs in almost one-third of human cancers, especially PC [36–42]. RAS is a common signaling component of both the MAPK and PI3K/AKT signaling pathways. And PDAC is the predominant form of PC. Recently, it has been reported that KRAS is the most frequently mutated gene (~95%) in PDAC [43, 44]. Consistent with previous research, in this study, there is a significant recruitment outcome for PI3K/AKT (for tDEGs) and MAPK signaling pathways (for tDEmiRs). Substantial efforts have been invested in developing the inhibitors of the PI3K/AKT and MAPK pathways; however, ascribed to complex crosstalk, clinical benefits are largely limited [43]. The interaction of RUNX2 and LAMC2 with the PI3K/AKT and MAPK signaling pathways can act as a driving force in controlling tumor progression [45, 46]. As a downstream molecule of the PI3K/AKT and MAPK pathway, RUNX2 can regulate metastatic properties of human prostate cancer and breast cancer cells [46–50]. However, RUNX2 also activates the PI3K/AKT pathway by regulating its components [51, 52]. Previous research has identified that the expression of LAMC2 depends largely on PI3K/AKT and MAPK pathways, and its overexpression also has a feedback regulation on these two pathways [53]. In non-small cell lung cancer cells, the inhibition of Akt upregulates LAMC2 expression, while high LAMC2 suppresses Akt signaling [53, 54]. Degen et al. found that MAPK/ERK pathway hyperactivation as the driver of LAMC2 overexpression by neoplastic cells, correlated with increased phosphorylation and activation of the translation factors S6 and eIF4B [55]. And overexpression of LAMC2 increased the protein expression of p38 but not ERK1/2, JNK or ERK5 [56]. In this study, we found that knockdown of LAMC2 or RUNX2 inhibited PI3K/AKT and MAPK/ERK pathways to a more or less extent, especially RUNX2. These may suggest directions for the discovery of the agents' combination that target PI3K/AKT and MAPK/ERK pathways in PC.

In summary, we confirmed that RUNX2 and LAMC2 are key genes that facilitate the progression of PC through bioinformatics and experimental analysis. Eight key miRNAs (miR-588, miR-199b, miR-1227, miR-628, miR-488, miR-1825, miR-519 and miR1265) which participated in the regulation of key genes expression could enrich the specific miRNA profiles of PC. These findings provide a new perspective on the underlying molecular mechanism of PC, suggesting that LAMC2 and RUNX2 may be valuable biomarkers and therapeutic targets for PC patients and may also offer powerful evidence and clues for the future genomic individualized treatment of PC.

## Supplementary Material

Supplementary Figures

Supplementary Tables
