# Supplementary material for: RUNX2 and LAMC2: promising pancreatic cancer biomarkers identified by an integrative data mining of pancreatic adenocarcinoma tissues
Source: Aging (Albany NY). 2021 Oct 4;13(19):22963–84. doi: 10.18632/aging.203589 (PMC8544338; doi:10.18632/aging.203589)
Supplement: Supplementary Figures [file aging-13-203589-s001.pdf]

SUPPLEMENTARY FIGURES

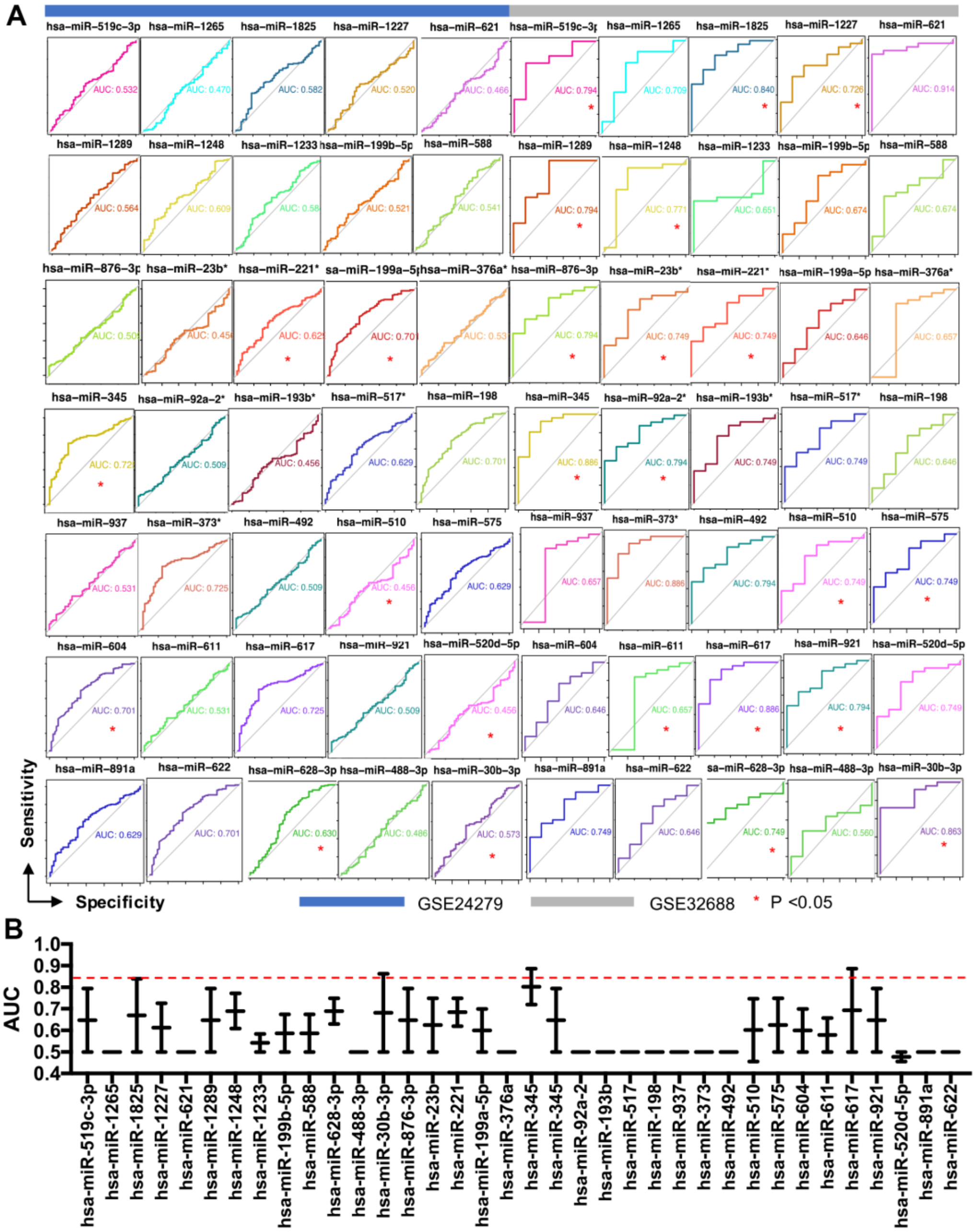

Supplementary Figure 1. ROC analysis of key genes related DEMs in GSE24279 and GSE32688. (**A**) ROC curve for 35 key genes related DEMs in GSE24279 and GSE32688. (**B**) Mean value of AUC results of 35 DEMs.

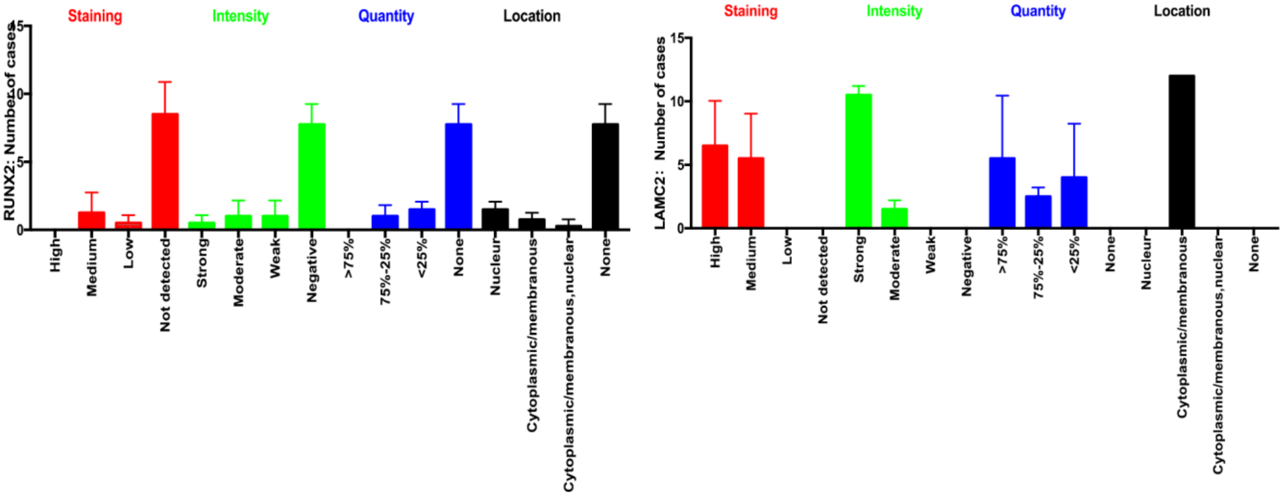

Supplementary Figure 2. The bar charts showed the summary (staining, intensity, quantity and location of RUNX2 and LAMC2) of IHC in PC patients.

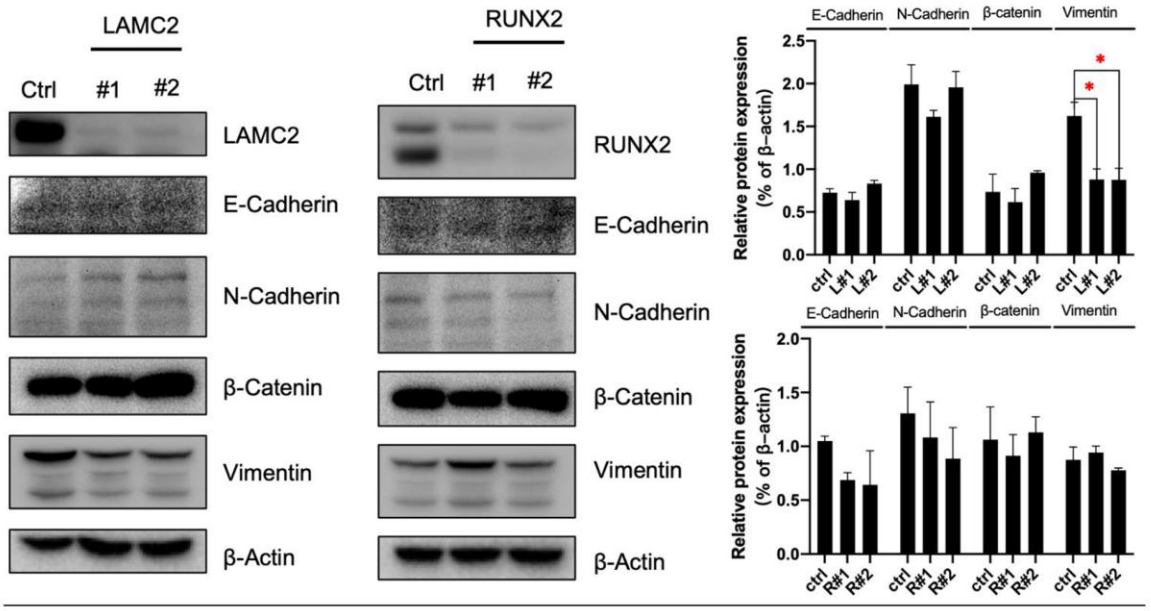

Supplementary Figure 3. ASPC-1 cells were stably transfected with control shRNA (NC) or two shRNAs of different sequences targeting LAMC2 or RUNX2; cell samples were collected and subjected to western blot analysis of E-Cadherin, N-Cadherin, β-catenin and vimentin.
