# Supplementary material for: RUNX2 and LAMC2: promising pancreatic cancer biomarkers identified by an integrative data mining of pancreatic adenocarcinoma tissues
Source: Aging (Albany NY). 2021 Oct 4;13(19):22963–84. doi: 10.18632/aging.203589 (PMC8544338; doi:10.18632/aging.203589)
Supplement: Supplementary Tables [file aging-13-203589-s002.pdf]

SUPPLEMENTARY TABLES

Supplementary Table 1. Combined OS analysis of RUNX2 and LAMC2 expression in TCGA PAAD patients.

Variables	MST (months)	HR (95%CI)	P value
Low RUNX2+Low LAMC2	19.66		
Low RUNX2+High LAMC2	16.37	1.194 (0.6816 to 2.092)	0.5169
High RUNX2+Low LAMC2	23.08	0.855 (0.5011 to 1.459)	0.5726
High RUNX2+High LAMC2	18.67	1.336 (0.7511 to 2.377)	0.2879

HR for the univariate Cox proportional hazards regression analysis.
Abbreviations: MST, median survival time; HR, hazard ratio; CI, confidence interval; PAAD, pancreatic adenocarcinoma.

Supplementary Table 2. Combined OS analysis of RUNX2 and LAMC2 expression in TCGA PAAD patients.

Variables	MST (months)	HR (95%CI)	P value
Low RUNX2+Low LAMC2	17.92		
Low RUNX2+High LAMC2	12.92	1.663 (0.9453 to 2.927)	**0.048**
High RUNX2+Low LAMC2	17.26	1.386 (0.8127 to 2.362)	0.1983
High RUNX2+High LAMC2	13.05	1.831 (1.002 to 3.347)	**0.021**

HR for the univariate Cox proportional hazards regression analysis.
Abbreviations: MST, median survival time; HR, hazard ratio; CI, confidence interval; PAAD, pancreatic adenocarcinoma.
